# The Substitution of Fish Meal or Chicken Meal With Yeast Culture in Diets of Bullfrogs (*Lithobates catesbeianus*): Growth Performance, Serum Biochemical Indices, Intestinal Digestive Enzyme, Hepatic Antioxidant, and Hepatic and Intestinal Histology

**DOI:** 10.1155/2024/2178697

**Published:** 2024-09-20

**Authors:** Yun-Feng Chen, Zhong-Chao Sun, Xu Yang, Yu-Fei Zheng, Yuan-Yuan Wang, Xiao-Qin Li, Xiang-Jun Leng

**Affiliations:** ^1^ National Demonstration Center for Experimental Fisheries Science Education Shanghai Ocean University, Shanghai, China; ^2^ Centre for Research on Environmental Ecology and Fish Nutrition of the Ministry of Agriculture and Rural Affairs Shanghai Ocean University, Shanghai, China; ^3^ Shanghai Collaborative Innovation for Aquatic Animal Genetics and Breeding Shanghai Ocean University, Shanghai, China; ^4^ Shanghai Yuanyao Biological Co. Ltd., Shanghai, China

**Keywords:** bullfrog, chicken meal, fish meal, growth, yeast culture

## Abstract

The aim of this study was to reveal the effects of yeast culture replacing fish meal (FM) or chicken meal (CM) on the growth performance, serum biochemical indexes, intestinal digestive enzyme activities, hepatic antioxidant, and hepatic and intestinal histology of bullfrog (*Lithobates catesbeianus*). The basal diet contained 100 g/kg FM and 100 g/kg CM, and then yeast culture was used to decrease FM or CM level to 75 and 50 g/kg with yeast culture inclusion of 32 and 64 g/kg, respectively, resulting in five groups of isonitrogenous diets (control, FM75, FM50, CM75, and CM50). A total of 450 bullfrogs (45.5 ± 0.4 g initial weight) were fed the five diets for 50 days. (1) The FM50 group presented significantly lower weight gain, condition factor, hind leg index, and higher feed conversion ratio than the control group, while the other three groups of FM75, CM75, and CM50 showed no significant difference in growth performance when compared to the control group. (2) The serum triglyceride content of FM50 group was significantly lower, while the alkaline phosphatase activity was significantly higher than those of the control group. The serum total cholesterol levels were significantly lower in the CM50 group compared to the control group. (3) In intestinal digestive enzyme activities, the trypsin and α-amylase activities in the CM75 and CM50 groups, the trypsin activity in the FM75 group, and lipase activity in the CM50 group were all significantly higher than those in the other groups. (4) The replacement of 50% FM with yeast culture (FM50 and CM50 groups) promoted the total antioxidant capacity in the liver, but compared to the control group, the intestinal villi height and muscularis propria thickness in the FM50 group were significantly lower. There was no difference (*P* > 0.05) in liver histology among all the groups. In conclusion, in a basal diet containing 100 g/kg FM and 100 g/kg CM, 32.0 and 64.0 g/kg yeast cultures could successfully replace 25% of dietary FM and 50% of dietary CM without negative effects on the growth performance, serum biochemical indexes, and hepatic and intestinal health of bullfrogs.

## 1. Introduction

Yeast culture (YC) is a complex fermentation product obtained by solid-state fermentation using *Saccharomyces cerevisiae* as the strain, which contains a variety of immunologically active substances such as β-glucan, mannan oligosaccharides (MOSs), cell-soluble substances, vitamins, proteins, and peptides [1]. It has been shown that the addition of 0.5% or 1.0% YC to feed significantly increased the ratio of intestinal villus height to crypt depth and the relative abundance of *Lactobacillus* in *Litopenaeus vannamei* [2]. The dietary addition of 4% YC significantly increased survival rate and weight gain (WG) of hybrid grouper (♀ *Epinephelus fuscoguttatus* × ♂ *E. lanceolatus*) [3]. Similar reports have also been found in largemouth bass (*Micropterus salmoides*) [4] and rainbow trout (*Oncorhynchus mykiss*) [5]. In addition, YC can also be used as protein source to partially replace fish meal (FM) inclusion in aquatic feeds. Bian et al. [6] replaced 100.0 g/kg FM with 131.4 g/kg YC (557.0 g/kg crude protein) in largemouth bass diet with 350 g/kg FM inclusion and found no differences (*P* > 0.05) in growth performance, nutrient utilization, antioxidant capacity, or intestinal health. It was reported by Duan [7] that 80.0 g/kg YC (557.0 g/kg crude protein) successfully substituted 59.0 g/kg FM in a basal diet containing 250.0 g/kg FM without negative effects on growth performance, nutrient utilization, intestinal histology, and microorganisms of hybrid snakehead (*Channa argus* ♂ × *Channa maculate* ♀). Huang et al. [8] and Zhang et al. [9] also reported the similar replacement of FM with YC in yellow catfish (*Pelteobagrus fulvidraco*) and gibel carp (*Carassius auratus gibelio* CAS Ⅲ).

Bullfrog (*Lithobates catesbeianus*) belongs to Chordata, amphibian, Anura, and Ranidae. For its tasty meat and rapid growth, bullfrog has become an important amphibian culture species in many countries, and worldwide consumption of bullfrog is increasing [10]. In commercial feed for bullfrogs, FM and chicken meal (CM) are important animal protein sources [11], which leads to the high cost of bullfrog feed. Currently, some alternative proteins have been reported to replace FM in bullfrog feeds, including soybean meal [12], fermented soybean meal [13, 14] and *Tenebrio molitor* meal [15]. But the substitution of YC for FM or CM has not been reported in bullfrogs. Therefore, this study investigated the effects of YC replacing different ratios of FM or CM on growth performance, muscle nutrient composition, serum biochemical indices, intestinal digestive enzyme activities, and liver antioxidant activity and liver and intestinal histological structure of bullfrogs. This study can provide a basis for the application of YC in bullfrog feed and the development of low animal protein bullfrog feed.

## 2. Materials and Methods

### 2.1. Ethical Statement

The study followed the animal care protocols set by the Institutional Animal Care and Use Committee and Shanghai Ocean University's Experimental Animal Ethics Committee (permit number: SFI 2021-24 of July 20, 2021).

### 2.2. Experimental Diets and Design

The basal diet contained 100 g/kg FM and 100 g/kg CM, and then YC was used to decrease FM or CM level to 75 and 50 g/kg with YC inclusion of 32 and 64 g/kg, respectively, resulting in five groups of isonitrogenous diets (control, FM75, FM50, CM75, and CM50). After ultra-microcrushing and screening through the 80-mesh sieve, all solid ingredients were weighed according to the formula (Table 1), then mixed, and added with oil and water. The mixture was pelleted by twin-screw extruder (TSE65, Beijing Modern Yanggong Machinery Technology Development Co., Ltd.) (pelletizing temperature of 110–125°C) to form floating feed with diameter of 4.0 mm and then dried at 60°C until the moisture content was less than 10%. All diets were sealed and stored in cool and dry place for use.

The YC were obtained from the Shanghai Yuanyao Biological Co., Ltd., which was produced through deep fermentation using enzymolysis soybean meal as substrate with the addition of glucose gluten and yeast (*S. cerevisiae*) (0.5 × 10^9^ CFU/g) [7]. The nutrient and amino acid compositions of YC are shown in Table 2.

### 2.3. Bullfrogs and Feeding Management

The bullfrogs were bought from Shantou Frog Fry Breeding Base in Guangdong Province, China. After 2 weeks of stocking with commercial diet (400 g/kg crude protein content), a total of 450 bullfrogs (average weight 45.5 ± 0.4 g) with healthy body and uniform size were selected for the feeding trial. A total of five treatments were set up with three replicates per treatment. The bullfrogs were randomly assigned to 15 cages (1.5 m × 1.5 m × 1.2 m, sieve 3 mm) with 30 bullfrogs per cage, which were hung in three concrete ponds (5.0 m × 3.0 m × 1.2 m). Considering the amphibious habit, about one-third of water area inside the cages was covered with floating plate to allow the bullfrogs to rest. The bullfrogs were hand-fed to satiation twice a day (07:30 and 16:30), and the feed amount was adjusted according to the weather, water temperature, and feeding response. The water was changed every 3 days, and the feces was siphoned out every 7 days. The water temperature and water depth were 26.3 ± 2.9°C and 28–30 cm, respectively. The feeding experiment was carried out in indoor pools under natural light at the Binhai Aquaculture Station of Shanghai Ocean University. The feeding period lasted for 50 days.

### 2.4. Sample Collection

After 24 h of fasting, the number of frogs in each cage was counted and weighed to calculate feed intake (FI), final body weight (FBW), WG, feed conversation ratio (FCR), and survival rate. Three bullfrogs were taken from each cage and executed according to the double marrow destruction method. The body length and weight were measured to calculate condition factor (CF). Then, the three bullfrogs were dissected, and blood was drawn from the aorta of heart with syringe (1 mL), left to stand for 12 h (4°C), and centrifuged at 2500 r/min for 10 min, and the supernatant was collected and stored at −80°C for the determination of serum biochemical indexes. The viscera, liver, and mesenteric fat were separated and weighed to calculate the viscerosomatic index (VSI), hepatosomatic index (HSI), and mesenteric fat index (MFI). A part of the jejunum (2 cm) and liver (1 cm × 0.5 cm × 0.5 cm) were sampled and fixed in Bouin's solution, and the rest of the intestine and liver were preserved at −80°C for the detection of intestinal digestive enzyme activities and hepatic antioxidant capacity. Finally, the hind legs of the bullfrogs were removed and then weighed for the calculation of the hind leg index (HLI), followed by storing the hind leg muscles at −80°C for the proximate composition analysis.

### 2.5. Indicators and Methods

#### 2.5.1. Growth and Body Indicators

The measurements or calculations of WG, FCR, survival rate, HSI, and HLI referred to Lin et al. [16]. FI, VSI, CF, and MFI measurements referred to Xu et al. [17] and Du et al. [18], respectively.

#### 2.5.2. Proximate Composition of Feed and Muscle

Moisture, crude protein, crude fat, and ash were determined by the methods described by AOAC [19]. Moisture and crude protein content were detected by oven-drying at 105°C to constant weight and an automatic Kjeldahl nitrogen tester (2300-Auto-Analyzer, Fosstecator, Sweden). Crude fat and ash contents were measured by chloroform–methanol extraction and scorching at 550°C in mufffe furnace (SXL-1008 Muffle Furnace, Shanghai Jinhong Experimental Equipment Co., Shanghai, China).

#### 2.5.3. Serum Biochemical Indicators

For the determination of triglyceride (TG) and total cholesterol (TCHO) contents, aspartate aminotransferase (AST), alanine aminotransferase (ALT), and alkaline phosphatase (AKP) activities, refer to Duan [7], while for lysozyme (LZM), refer to Bian et al. [6].

#### 2.5.4. Indicators Related to Intestinal Digestion and Hepatic Antioxidants

Samples of the intestines and livers were weighed, and nine times volume of saline was added and then mechanically homogenized in an ice-water bath, centrifuged at 10,000 r/min for 10 s (four times), and followed by centrifugation at 3000 r/min for 10 min. The supernatant was collected and diluted with saline (1:9) to form 1% tissue homogenate, which was used for the determination of enzyme activities. Intestinal trypsin, lipase, and amylase activities were determined with reference to Wang et al. [3]. The measurement of liver malondialdehyde (MDA) content, total superoxide dismutase (T-SOD), and catalase (CAT) activities referred to Chen et al. [20], while total antioxidant capacity (T-AOC) referred to Randall et al. [21]. All the kits for the determination of the above indexes were supplied by Nanjing Jiancheng Bioengineering Institute.

#### 2.5.5. Liver and Intestinal Histomorphology

The process includes sampling, dehydration, transparency, wax dipping, embedding, sectioning (5–6 μm, LEICA RM2145), staining, sealing, and microscopic observation (Olympus DP71) [7].

### 2.6. Statistical Analysis

All data were expressed as mean ± standard deviation. One-way ANOVA was performed using SPSS 25.0 where Tukey's was used for multiple comparisons. The *P*-value less than 0.05 represents statistical significance.

## 3. Results

### 3.1. Growth Performance

After 50 days of culture, all the bullfrogs presented good growth with 100% survival rate and reached the commercial size (about 200 g). There was no difference (*P* > 0.05) in FBW, WG, FCR, CF, and MFI among the four groups of control, FM75, CM75, and CM50. The FM50 group showed a significant decrease in the above indicators and increase in FCR and MFI compared to the control group. There was no difference (*P* > 0.05) in FI, VSI, and HSI among all the groups (Table 3).

### 3.2. Muscle Proximate Composition

There was no difference (*P* > 0.05) in muscle proximate composition such as crude protein, crude fat, moisture, and ash contents between the alternative groups and the control group (Table 4). The CM50 group showed significantly higher crude fat level than the FM 75 and FM 50 groups (*P* < 0.05).

### 3.3. Serum Biochemical Indicators

With the increase of YC replacing FM and CM levels, serum AKP and LZM activities tended to increase, and TG and TCHO levels tended to decrease. Serum TG contents in FM50 group and TCHO in CM50 group were significantly lower, while serum AKP activity in FM50 and CM50 groups and LZM activity in CM75 group were significantly higher than those in the control group. There was no difference (*P* > 0.05) in AST and ALT activities between the alternative groups and the control group (Table 5).

### 3.4. Liver Antioxidant Capacity and Histology

In Table 6, T-AOC in the FM75, FM50, and CM50 groups was significantly higher, and MDA content in the CM75 group was significantly lower than those in the control group. There was no difference (*P* > 0.05) in T-SOD and CAT activities between the alternative groups and the control group.

**Figure 1:**
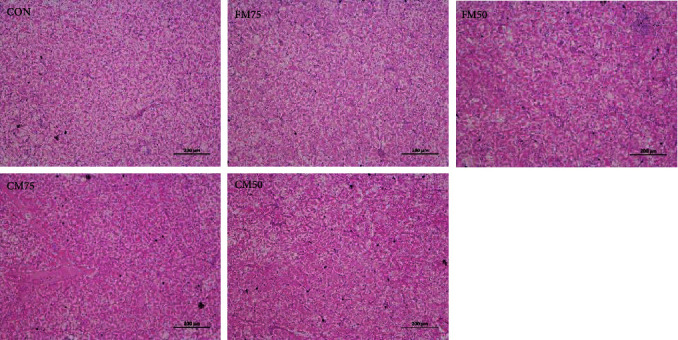
Effects of fish meal or chicken meal replacement by yeast culture on liver tissue structure of bullfrog.

As shown in Figure 1, all the groups presented the similar liver morphology with intact hepatocytes structure. The cells were clearly visible with oval or polygonal shape and clear boundaries. No morphological difference was observed among all the groups.

### 3.5. Intestinal Digestive Enzyme Activity and Histology

Intestinal trypsin activity in the FM75, CM75, and CM50 groups and α-amylase activity in the CM75 and CM50 groups were significantly higher than those in the control group. Intestinal lipase activity was significantly increased in all replacement groups, when compared to the control group. Specifically, the CM50 group had the highest lipase activity (*P* < 0.05) (Table 7).

**Figure 2:**
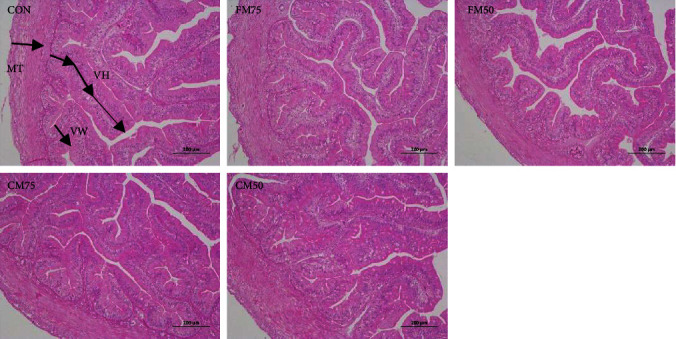
Effects of fish meal or chicken meal replacement by yeast culture on intestinal tissue structure of bullfrog. MT, muscularis thickness; VH, villus height; VW, villus width.

As shown in Figure 2 and Table 8, the FM50 group presented incomplete intestinal structure with significantly lower intestinal villi height and width (VH and VW) (*P* < 0.05), while the CM50 group had significantly higher muscularis thickness (MT) than the control group (*P* < 0.05).

## 4. Discussion

### 4.1. Growth Performance

YC contains plenty of protein, small peptides, nucleotides, MOS, β-glucan, and unknown growth factors [1, 22]. In this study, replacing 25.0 g/kg FM with 32.0 g/kg YC did not reduce the growth performance in bullfrogs. Similarly, Zhang et al. [9] successfully substituted 40.0 g/kg FM with 40 g/kg YC (crude protein 667.25 g/kg) without negative effects on the growth performance of gibel carp. Successfully partial replacement of FM by YC has also been reported in Pacific white shrimp [23], largemouth bass [6], hybrid snakehead [7], and yellow catfish [8]. It is noteworthy that the present replacement of 50.0 g/kg FM (50% of dietary FM) with 64.0 g/kg YC resulted in a significant decrease in WG, CF, and HLI and a significant increase in FCR and MFI, which may be attributed to the relative imbalance in amino acid composition of YC (e.g., low methionine level) (Table 2), the lack of certain active substances (e.g., taurine), and high fiber content [7].

However, the replacement of 50% CM (CM50) by YC did not negatively affect growth performance of bullfrogs compared to replacement of 50% FM, even presented significantly higher crude fat content than the FM 75 and FM 50 groups. In hybrid snakehead, the substitution of 80.0 g/kg CM with 80.0 g/kg YC (557.0 g/kg crude protein) also showed no negative effects on growth performance [7]. Such results indicated that YC has higher substitutability for CM and higher potential for fat deposition than FM. In bullfrog feed, can YC replace all dietary CM? Or what is the upper limit of replacing CM? To answer the question, more studies are needed in the future.

### 4.2. Serum Biochemical Indicators

In hybrid snakehead [7] and Pacific white shrimp [24], serum TG and TCHO contents showed a decreasing trend with the replacement of FM by 75.0 g/kg and 80.0 g/kg YC, respectively. In the present study, the serum TG (FM50) and the TCHO content (CM50) were significantly reduced by the replacement of FM and CM with YC. It has been reported that YC could accelerate fat metabolism [25]. Jayachandran et al. [26] found that β-glucan in YC effectively reduced serum TCHO content. In addition, serum CHO content is influenced by CHO contents in feed ingredients [27]. FM and CM are animal protein sources rich in CHO, while the fermentation substrate of YC is soybean meal (plant protein source) without cholesterol inclusion. Therefore, serum TCHO level decreased as YC replaced high level of FM or CM.

Serum AST and ALT activities would rise when the liver of animal is injured [28]. In largemouth bass, the replacement of 150.0 g/kg FM with YC did not produce significant effect on serum AST and ALT activities [6]. The replacement of 88.5 g/kg FM, or 80.0 g/kg CM with YC also showed no significant effects on serum AST and ALT activities in hybrid snakehead [7]. No differences (*P* > 0.05) were observed in serum AST and ALT activities and liver histological when partial FM were substituted in the present study.

LZM and AKP, as innate immune indicators, can be used for nonspecific immune detection in organisms [29]. Serum LZM activity of largemouth bass was significantly promoted by the replacement of FM (150.0 g/kg) with YC (197.1 g/kg) [6]. In the present study, serum AKP increased significantly, and LZM activity tended to increase when YC replaced 50% FM or CM. The modulated immunity by dietary YC may be related to the fact that YC is rich in MOS and β-glucan [24, 29]. Ren et al. [30] found that dietary addition of 20.0 g/kg MOS increased serum LZM activity in hybrid grouper (*Epinephelus lanceolatus* ♂ × *E. fuscoguttatus* ♀). The addition of 2.0 g/kg MOS to feed increased serum LZM and AKP activities in *Anguilla anguilla* [31]. Some studies have shown that MOS and β-glucan can directly bind to leukocytes and macrophages and then activate them to release interleukins and cytokines etc., enhancing the immunity of organism [26, 32].

### 4.3. Liver Antioxidant

Yan et al. [33] found that the addition of 0.06% YC to feed significantly increased the CAT activity in the liver of mirror carp (*Cyprinus carpio* var. *specularis* ”Songpu”). In Pacific white shrimp, dietary YC (0.3%, 0.5%, or 1.0%) also significantly increased hepatopancreatic CAT and SOD activities [34]. In this study, the increasing replacement of FM or CM with YC tended to promote TAOC and decrease MDA content in the liver of bullfrogs, reflecting that YC may be beneficial in improving the antioxidant capacity of bullfrogs. The possible reason is that YC is rich in MOS, β-glucan, etc. [7]. β-glucan has the potential to enhance the organism's antioxidant capacity through the scavenging of free radicals and the upregulation of antioxidant enzyme gene expression [35, 36]. Dietary MOS (0.5%) has been found to significantly increase liver SOD, CAT, and glutathione peroxidase activities in the liver of tilapia (*Oreochromis niloticus*) [37]. In addition, the active peptides and some unknown factors in YC may contribute to the antioxidant capacity improvement of the organism.

### 4.4. Intestinal Digestive Enzyme Activity and Histology

The increasing activities of intestinal trypsin, lipase, and α-amylase can reflect the enhancement of digestive ability [7]. In hybrid grouper, the substitution of 15 or 30 g/kg FM with YC (500 g/kg crude protein) significantly increased intestinal trypsin, lipase, and α-amylase activities [38]. In this study, the intestinal trypsin, lipase, and α-amylase activities of bullfrogs were increased when YC replaced 25.0 g/kg FM or 50.0 g/kg CM. Normally, some organic acids will be produced during the yeast fermentation, such as lactic acid. It has been reported that dietary lactic acid can increase the intestinal trypsin activity of grass carp (*Ctenopharyngodon idellus*) [39] and α-amylase activity of hybrid tilapia (*O. niloticus* × *O. aureus*) [40].

Intestinal villi facilitate the absorption of nutrients and help to promote the growth of the organism [41]. The intestinal VH and VW determine the contact area of intestinal mucosal epithelial cells with the chyme, whereas an increase in the intestinal MT facilitates intestinal digestion [42]. Yuan et al. [43] reported that substituting 52.5 g/kg FM with 70 g/kg YC significantly improved the intestinal VH of Jian carp (*C. carpio* var. Jian carpio) juvenile. In grouper [38], the substitution of 15 or 30 g/kg FM with YC (500 g/kg crude protein) also significantly increased the VH and VW of intestinal tract. In this experiment, the replacement of 50% FM with YC (CM50 group) significantly increased the intestinal MT. The improvement of intestinal tissue morphology by YC may be related to the presence of nucleotides as well as MOS in YC [22]. Nucleotides and MOS may protect the structural integrity of the gut [44]. It has been found that nucleotides increased the intestinal MT [45]. MOS can increase the utilization of fat by stimulating the organism to secrete intestinal mucus [46]. As reported by Zhang et al. [47], Pacific white shrimp's intestinal VH and absorption area were improved significantly when MOS was added to feed at 2.0 g/kg. According to Dimitroglou et al. [44], dietary addition of 4 g/kg MOS significantly increased intestinal VH and absorptive area of gilthead seabream (*Sparus aurata*). However, the replacement of 50% FM with YC significantly decreased intestinal VH and VW of hybrid snakehead, which may be due to the reduced taurine levels in high YC diet [7]. In black carp (*Mylopharyngodon piceus*), dietary supplementation of 0.5 g/kg taurine significantly increased the intestinal VH of fish fed with low FM diet (10%) [48].

## 5. Conclusion

In a basal diet containing 100 g/kg of FM and 100 g/kg CM, 32.0 g/kg and 64.0 g/kg YC could successfully replace 25.0 g/kg FM and 50.0 g/kg CM without negative effects on the growth performance and hepatic and intestinal health of bullfrogs.

## Figures and Tables

**Table 1:** Composition and nutrient levels of experimental diets (air-dry basis, g/kg).

Ingredients^a^	Control	FM75	FM50	CM75	CM50
Fish meal	100.0	75.0	50.0	100.0	100.0
Yeast culture	0.0	32.0	64.0	32.0	64.0
Chicken meal	100.0	100.0	100.0	75.0	50.0
Pork powder	50.0	50.0	50.0	50.0	50.0
Soybean protein concentrate	50.0	50.0	50.0	50.0	50.0
Corn gluten	60.0	60.0	60.0	60.0	60.0
Soybean meal	290.0	290.0	290.0	290.0	290.0
Wheat flour	280.0	273.0	266.0	273.0	266.0
Fish oil	10.0	10.0	10.0	10.0	10.0
Soybean oil	10.0	10.0	10.0	10.0	10.0
Soybean phospholipid	15.0	15.0	15.0	15.0	15.0
Monocalcium phosphate	20.0	20.0	20.0	20.0	20.0
Choline chloride	5.0	5.0	5.0	5.0	5.0
Vitamin and mineral premixes^b^	10.0	10.0	10.0	10.0	10.0
Total	1000.0	1000.0	1000.0	1000.0	1000.0
Proximate composition					
Crude protein	405.6	410.0	403.2	407.1	403.0
Crude fat	72.0	70.5	69.0	69.9	69.7
Crude ash	72.7	71.8	69.8	69.6	70.2
Moisture	66.7	64.6	64.5	66.4	66.3

^a^The crude protein contents of fishmeal, yeast culture, chicken meal, pork powder, soy protein concentrate, corn protein meal, soybean meal, and wheat flour were 700.5, 550.0, 655.0, 680.0, 618.8, 590.0g, 466.2, and 140.0 g/kg, respectively.

^b^VA, 800,000 IU/kg; VD_3_, 200,000 IU/kg; VE, 9000 mg/kg; VK_3_, 1500 mg/kg; VB_1_, 1200 mg; VB_2_, 2400 mg; VB_6_, 1000 mg; VB_12_, 12 mg; calcium pantothenate, 4400 mg; nicotinamide, 5800 mg; folic acid, 570 mg; biotin, 20 mg; inositol, 9500 mg; VC, 20,000 mg; L-carnitine, 2000 mg; iron, 20 g; copper, 1.2 g; zinc, 12.5 g; manganese, 6 g; magnesium, 8 g; iodine, 400 mg; selenium, 45 mg; cobalt, 150 mg.

**Table 2:** Proximate composition and amino acid composition of yeast culture (air-dry basis, g/kg) [7].

Items	Content (g/kg)
Nutrient composition	
Crude protein	557.0
Crude fat	16.0
Mannan oligosaccharides	22.0
Nucleotide	2.01
Amino acid composition	
Phenylalanine	30.2
Threonine	22.3
Valine	25.5
Leucine	43.1
Lysine	34.8
Methionine	8.6
Arginine	39.8
Histidine	15.1
Isoleucine	25.4
Nonessential amino acid	
Serine	27.7
Tyrosine	18.1
Glycine	21.8
Proline	27.7
Cysteine	8.0
Glutamic acid	92.4
Alanine	23.8
Aspartic acid	56.4
Total amino acid	520.7

**Table 3:** Effects of fish meal or chicken meal replacement by yeast culture on growth performance of bullfrog.

Items	Control	FM75	FM50	CM75	CM50
IBW (g)	45.7 ± 0.5	45.7 ± 0.3	45.7 ± 0.4	45.6 ± 0.2	45.2 ± 0.6
FBW (g)	206.2 ± 2.4^a^	206.2 ± 2.3^a^	184.6 ± 12.2^b^	213.0 ± 12.3^a^	211.9 ± 3.4^a^
WG (%)	351.5 ± 9.1^a^	350.9 ± 15.5^a^	291.8 ± 2.4^b^	366.5 ± 26.9^a^	369.1 ± 13.0^a^
FCR	1.11 ± 0.02^b^	1.11 ± 0.05^b^	1.35 ± 0.02^a^	1.07 ± 0.08^b^	1.07 ± 0.03^b^
FI (g/frog)	178.15 ± 0.26	178.07 ± 0.16	177.86 ± 0.23	177.62 ± 0.36	177.78 ± 0.52
Survival rate (%)	100.0 ± 0.0	100.0 ± 0.0	100.0 ± 0.0	100.0 ± 0.0	100.0 ± 0.0
CF (g/cm)^3^	10.36 ± 0.20^a^	10.64 ± 0.55^a^	8.86 ± 0.13^b^	10.42 ± 0.56^a^	10.46 ± 0.68^a^
HLI (%)	39.70 ± 0.24^a^	38.22 ± 0.87^abc^	36.68 ± 0.06^c^	38.00 ± 0.61^bc^	38.64 ± 0.97^ab^
VSI (%)	20.05 ± 1.41	20.76 ± 1.25	19.16 ± 1.40	19.23 ± 1.38	18.64 ± 0.91
HSI (%)	4.33 ± 0.24	4.22 ± 0.24	4.43 ± 0.30	4.45 ± 0.36	4.49 ± 0.44
MFI (%)	4.58 ± 0.29^b^	4.68 ± 0.15^b^	5.47 ± 0.41^a^	4.54 ± 0.30^b^	4.40 ± 0.35^b^

*Note:* In the same row, values with different letter superscripts indicate significant differences (*P*  < 0.05).

Abbreviations: CF, condition factor; FBW, final body weight; FCR, feed conversion ratio; FI, feed intake; HLI, hind leg index; HSI, hepatosomatic index; IBW, initial body weight; MFI, mesenteric fat index; VSI, viscerosomatic index; WG, weight gain.

**Table 4:** Effects of fish meal or chicken meal replacement by yeast culture on flesh composition of bullfrog (fresh tissue, g/kg).

Items	Control	FM75	FM50	CM75	CM50
Moisture	794.3 ± 6.7	795.2 ± 8.8	785.4 ± 1.7	784.9 ± 3.4	788.5 ± 5.7
Crude protein	180.8 ± 4.5	182.3 ± 4.0	184.7 ± 1.1	185.5 ± 1.8	184.4 ± 6.2
Crude fat	7.1 ± 0.8^ab^	5.9 ± 0.5^b^	6.5 ± 0.4^b^	7.0 ± 0.4^ab^	8.6 ± 0.5^a^
Ash	9.3 ± 0.5	8.5 ± 0.1	9.6 ± 0.1	9.3 ± 0.2	8.8 ± 0.4

*Note:* In the same row, values with different letter superscripts indicate significant differences (*P* < 0.05).

**Table 5:** Effects of fish meal or chicken meal replacement by yeast culture on serum biochemical indicators of bullfrog.

Items	Control	FM75	FM50	CM75	CM50
TG (mmol/L)	1.98 ± 0.15^a^	1.73 ± 0.19^ab^	1.42 ± 0.15^b^	1.62 ± 0.18^ab^	1.51 ± 0.06^ab^
TCHO (mmol/L)	3.26 ± 0.35^ab^	3.60 ± 0.31^a^	2.40 ± 0.36^bc^	2.74 ± 0.23^ab^	1.68 ± 0.15^c^
AST (U/mL)	2.78 ± 0.16^ab^	3.09 ± 0.11^a^	2.98 ± 0.12^ab^	2.49 ± 0.21^b^	2.67 ± 0.21^ab^
ALT (U/mL)	2.23 ± 0.18	1.85 ± 0.22	2.05 ± 0.30	1.78 ± 0.24	1.40 ± 0.19
AKP (Au/100 mL)	13.06 ± 0.23^c^	15.70 ± 0.34^bc^	17.43 ± 0.98^ab^	14.45 ± 0.69^c^	19.10 ± 1.50^a^
LZM (μg/mL)	6.36 ± 0.76^b^	6.91 ± 0.66^ab^	6.80 ± 0.68^ab^	8.06 ± 0.23^a^	8.00 ± 0.19^ab^

*Note:* In the same row, values with different letter superscripts indicate significant differences (*P* < 0.05).

Abbreviations: AKP, alkaline phosphatase; ALT, alanine aminotransferase; AST, aspartate aminotransferase; LZM, lysozyme; TCHO, total cholesterol; TG, triglycerides.

**Table 6:** Effects of fish meal or chicken meal replacement by yeast culture on liver antioxidant capacity of bullfrog.

Items	Control	FM75	FM50	CM75	CM50
T-SOD (U/mgprot)	126.31 ± 12.38	125.48 ± 2.31	125.71 ± 6.83	133.34 ± 7.48	139.78 ± 10.50
CAT (U/mgprot)	30.88 ± 2.29^ab^	25.00 ± 1.45^b^	37.01 ± 2.36^a^	32.30 ± 2.30^ab^	36.38 ± 3.36^a^
T-AOC (mmol/mL)	3.17 ± 0.29^c^	4.55 ± 0.33^ab^	5.29 ± 0.10^a^	4.04 ± 0.23^bc^	5.42 ± 0.42^a^
MDA (nmol/mgprot)	1.94 ± 0.05^a^	1.86 ± 0.25^a^	1.48 ± 0.16^ab^	1.28 ± 0.26^b^	1.45 ± 0.15^ab^

*Note:* In the same row, values with different letter superscripts indicate significant differences (*P* < 0.05).

Abbreviations: CAT, catalase; MDA, malonaldehyde; T-AOC, total antioxidant capacity; T-SOD, total superoxide dismutase.

**Table 7:** Effects of fish meal or chicken meal replacement by yeast culture on intestinal digestive enzyme activity of bullfrog.

Items	Control	FM75	FM50	CM75	CM50
Trypsin (U/mg prot)	2635.24 ± 93.03^b^	4491.63 ± 213.20^a^	3011.78 ± 325.31^b^	3914.65 ± 182.72^a^	4597.34 ± 83.01^a^
Lipase (U/g prot)	96.71 ± 3.95^c^	127.21 ± 5.99^b^	116.95 ± 5.00^b^	127.57 ± 1.10^b^	157.63 ± 13.71^a^
Amylase (U/mg prot)	0.20 ± 0.02^b^	0.24 ± 0.01^ab^	0.24 ± 0.02^ab^	0.27 ± 0.02^a^	0.30 ± 0.03^a^

*Note:* In the same row, values with different letter superscripts indicate significant differences (*P* < 0.05).

**Table 8:** Effects of fish meal or chicken meal replacement by yeast culture on intestinal morphology of bullfrog (μm).

Items	Control	FM75	FM50	CM75	CM50
VH	1717.5 ± 180.2^a^	2083.7 ± 118.3^a^	1372.5 ± 72.8^b^	1740.2 ± 128.0^a^	1826.2 ± 102.0^a^
VW	208.1 ± 4.4^a^	233.7 ± 15.9^a^	168.0 ± 7.4^b^	221.3 ± 16.9^a^	236.3 ± 19.0^a^
MT	184.2 ± 2.0^bc^	177.3 ± 4.8^c^	178.4 ± 14.1^c^	213.1 ± 5.0^b^	252.4 ± 5.2^a^

*Note:* In the same row, values with different letter superscripts indicate significant differences (*P* < 0.05).

Abbreviations: MT, muscularis thickness; VH, villus height; VW, villus width.

## Data Availability

The data that support the findings of this study are available from the corresponding author upon reasonable request.
